# Genome-wide investigation of NLP gene family members in alfalfa (*Medicago sativa* L.): evolution and expression profiles during development and stress

**DOI:** 10.1186/s12864-023-09418-x

**Published:** 2023-06-13

**Authors:** Jinqiu Yu, Yuying Yuan, Linling Dong, Guowen Cui

**Affiliations:** Department of Grassland Science, College of Animal Science and Technology, Northeast Agricultural University, Harbin, 150030 China

**Keywords:** *Medicago sativa*, NLP transcription factors, Genome-wide, Abiotic stress, Phytohormone

## Abstract

**Background:**

NIN-like protein (NLP) transcription factors (TFs) compose a plant-specific gene family whose members play vital roles in plant physiological processes, especially in the regulation of plant growth and the response to nitrate-nitrogen. However, no systematic identification or analysis of the NLP gene family has been reported in alfalfa. The recently completed whole-genome sequence of alfalfa has allowed us to investigate genome-wide characteristics and expression profiles.

**Results:**

53 *MsNLP* genes were identified from alfalfa and renamed according to their respective chromosome distributions. Phylogenetic analysis demonstrated that these *MsNLPs* can be classified into three groups on the basis of their conserved domains. Gene structure and protein motif analyses showed that closely clustered *MsNLP* genes were relatively conserved within each subgroup. Synteny analysis revealed four fragment duplication events of *MsNLPs* in alfalfa. The ratios of nonsynonymous (Ka) and synonymous (Ks) substitution rates of gene pairs indicated that the *MsNLP* genes underwent purifying selection during evolution. Examination of the expression patterns of different tissues revealed specific expression patterns of the *MsNLP* genes in the leaves, indicating that these genes are involved in plant functional development. Prediction of cis-acting regulatory elements and expression profiles further demonstrated that the *MsNLP* genes might play important roles in the response to abiotic stress and in phytohormone signal transduction processes.

**Conclusion:**

This study represents the first genome-wide characterization of *MsNLP* in alfalfa. Most *MsNLPs* are expressed mainly in leaves and respond positively to abiotic stresses and hormonal treatments. These results provide a valuable resource for an improved understanding of the characteristics and biological roles of the *MsNLP* genes in alfalfa.

**Supplementary Information:**

The online version contains supplementary material available at 10.1186/s12864-023-09418-x.

## Introduction

NIN-like proteins (NLPs) have been found in many species, such as those of green algae and slime molds, and are considered to belong to a plant-specific family of transcription factors (TFs) [1–4]. On the basis of previous researchers’ comparisons of NLP gene family members in different species, NLP TFs contain two typical characteristic domains: a highly conserved RWP-RK domain and a PB1 domain located at the C- terminus [5, 6]. The RWP-PK domain is highly conserved and consists of more than 50 amino acids, including characteristic “RWPXRK” residues. The PB1 domain is also highly conserved and consists of approximately 80 amino acids at the C-terminus of the NLP. The RWP-RK domain functions as a DNA-binding domain and alone can bind to nitrate-responsive cis-acting elements independently of nitrate [7, 8]. The PB1 domain is involved in protein–protein interactions by forming a dimer with other genes containing this domain, although some PB1 domains mediate interactions with proteins lacking the PB1 domain [9]. In addition, a highly conserved cGMP phosphodiesterase (GAF) domain was identified at the N-terminus of some NLPs and may be related to signal transduction or dimerization [5, 6].

The earliest research on NLP TFs can be traced back to the legume model plant species *Lotus japonicus Nin* (nodule inception). First, *Nin* was used as a nodule-sensing gene and was identified as affecting the early development of nodules [10]. Subsequently, a large number of *Nin* homologous *Nin-like* genes were identified in nonlegume plant species, including *Arabidopsis* [2], *rice* [5], *wheat* [11], *maize* [3] and *Brassica napus* [12]. In-depth research on NLP TFs has revealed the various domains, regulatory networks and expression patterns of the members of this family to a certain extent. Many studies have confirmed that NLP TFs play important roles in the response to nitrate-nitrogen and in plant growth and development. For example, *AtNLP7* functions in nitrate and nitrogen starvation responses by binding to key nitrogen pathway-related genes, including *ANR1*, *NRT1.1*, *NRT2*, and *LBD37/38*, thus moderating nitrogen assimilation and metabolism by transcriptionally activating or suppressing the expression of downstream genes [13, 14]. *AtNLP8* regulates nitrate-promoted seed germination and directly binds to the promoter of an abscisic acid (ABA) catabolism-related enzyme-encoding gene to reduce ABA levels in a nitrate-dependent manner [15]. The expression of Arabidopsis *NLP7* can promote plant growth by increasing the assimilation of nitrogen and carbon under both limited and sufficient nitrogen conditions [16]. NLP TFs are induced in response to abiotic stress and participate in the regulation of plant tolerance to abiotic stress. For instance, *AtNLP7* knockout plants were more drought resistant than wild- type plants. Therefore, NLP TFs not only play a very important role in regulating nitrogen absorption and assimilation but are also related to plant drought resistance [17]. In addition, it has been confirmed that NLP TFs regulate nitrate-responsive gene expression not only to promote nitrate uptake but also to modulate nodulation [18, 19].

Alfalfa (*Medicago sativa* L.) is an economically and ecologically important legume herbaceous species that is versatile, is highly productivity, has high feed value and plays potential roles in soil improvement and soil conservation [20–22]. So far the basic knowledge of the *NLPs* is still limited in alfalfa. Because *NLP* genes are involved in regulating various important physiological processes, it would be highly important to systematically investigate NLP family members in alfalfa. With the release of the whole-genome sequence of alfalfa, we have an opportunity to systematically investigate the evolutionary traits, organization and expression profiles of the NLP gene family members of alfalfa at the genome-wide level [23]. Our results provide insights into the evolutionary history and potential functional diversity of *NLP* genes in alfalfa.

## Results

### Identification of the NLP genes in the alfalfa genome

Searching for the RWP-RK conserved domain (PF02042) via an HMM profile, we identified a total of 53 putative NLP family genes in alfalfa, which we named *MsNLP1* to *MsNLP53*. The gene characteristics as well as the chromosome locations and the protein sequence length, molecular weight (MW), isoelectric point (pI), and subcellular locations were shown in Table 1. The lengths of the 53 identified *NLPs* ranged from 208 to 1312, their MWs ranged from 24.2 to 143.1 kDa, and their pIs ranged from 4.94 to 8.84. The 53 *MsNLP* genes were unevenly distributed on chromosomes 1 to 8 (Additional file 1). The largest number of genes (24) was found on chromosome 3, while only 1 *MsNLP* gene was distributed on chromosome 1. The predicted subcellular localization results showed that most of the *MsNLPs* were localized in the nucleus, and the others were localized in the mitochondria, chloroplasts and cytoplasm, the findings of which were comparable to those of *NLPs* from other plant species.

**Table 1:** Characterization of *NLP* genes in alfalfa

Gene name	Sequence ID	Chromosome location	Gene location	Protein length(aa)	MW (kDa)	pI	Predicted localization
*MsNLP01*	*MS.gene006277.t1*	chr2.1	5193880:5200037	979	108.76	5.45	Nucleus
*MsNLP02*	*MS.gene34150.t1*	chr1.2	75194857:75198598	899	99.72	5.81	Mitochondrion,Nucleus
*MsNLP03*	*MS.gene49108.t1*	chr1.3	69109302:69113709	913	101.28	5.67	Mitochondrion,Nucleus
*MsNLP04*	*MS.gene061167.t1*	chr1.4	77967430:77971158	898	99.47	5.83	Mitochondrion,Nucleus
*MsNLP05*	*MS.gene056973.t1*	chr2.1	5193880:5200037	993	109.44	5.64	Nucleus
*MsNLP06*	*MS.gene051987.t1*	chr2.2	3555733:3567676	1312	143.1	6.1	Nucleus
*MsNLP07*	*MS.gene032204.t1*	chr2.3	4245362:4251523	993	109.38	5.64	Nucleus
*MsNLP08*	*MS.gene85257.t1*	chr2.4	4833475:4839636	1021	112.81	5.65	Nucleus
*MsNLP09*	*MS.gene018926.t1*	chr3.1	28342850:28344703	294	34.72	5.95	Nucleus
*MsNLP10*	*MS.gene05746.t1*	chr3.1	48889662:48892282	550	62.29	5.47	Nucleus
*MsNLP11*	*MS.gene057117.t1*	chr3.1	73634592:73636487	266	31.01	6.19	Chloroplast, Nucleus
*MsNLP12*	*MS.gene057118.t1*	chr3.1	73639809:73640877	271	31.48	8.84	Chloroplast
*MsNLP13*	*MS.gene057118.t1*	chr3.1	73639809:73640877	500	56.41	5.11	Nucleus
*MsNLP14*	*MS.gene070016.t1*	chr3.1	91323675:91327682	909	101.89	5.63	Nucleus
*MsNLP15*	*MS.gene93356.t1*	chr3.2	26831787:26833416	294	34.71	5.82	Nucleus
*MsNLP16*	*MS.gene03941.t1*	chr3.2	55512939:55515559	555	62.72	5.51	Nucleus
*MsNLP17*	*MS.gene06908.t1*	chr3.2	77118645:77120065	208	24.2	5.79	Nucleus
*MsNLP18*	*MS.gene06906.t1*	chr3.2	77131484:77132908	237	27.54	7.02	Chloroplast, Nucleus
*MsNLP19*	*MS.gene06905.t1*	chr3.2	77136208:77137730	300	34.83	7.65	Chloroplast
*MsNLP20*	*MS.gene014987.t1*	chr3.2	86569930:86571992	500	56.35	5.19	Nucleus
*MsNLP21*	*MS.gene014342.t1*	chr3.2	91210898:91215452	909	101.95	5.59	Nucleus
*MsNLP22*	*MS.gene048526.t1*	chr3.3	52619183:52621780	550	62.32	5.52	Nucleus
*MsNLP23*	*MS.gene06695.t1*	chr3.3	76442776:76444715	266	30.99	6.19	Chloroplast, Nucleus
*MsNLP24*	*MS.gene06694.t1*	chr3.3	76447534:76449062	300	34.83	7.65	Chloroplast
*MsNLP25*	*MS.gene42693.t1*	chr3.3	86243303:86245386	500	56.41	5.15	Nucleus
*MsNLP26*	*MS.gene69323.t1*	chr3.3	94133992:94138543	909	101.93	5.59	Nucleus
*MsNLP27*	*MS.gene064839.t1*	chr3.3	94191629:94195674	909	101.89	5.63	Nucleus
*MsNLP28*	*MS.gene03560.t1*	chr3.4	30317366:30319242	290	34.18	5.82	Nucleus
*MsNLP29*	*MS.gene008665.t1*	chr3.4	60167964:60170583	551	62.35	5.38	Nucleus
*MsNLP30*	*MS.gene013578.t1*	chr3.4	83852409:83854343	266	31.09	6.19	Chloroplast, Nucleus
*MsNLP31*	*MS.gene013577.t1*	chr3.4	83867447:83868975	302	35.05	7.65	Chloroplast
*MsNLP32*	*MS.gene42616.t1*	chr3.4	91797537:91799567	488	55.04	5.11	Nucleus
*MsNLP33*	*MS.gene056401.t1*	chr4.1	33857982:33861940	917	102.13	5.16	Nucleus
*MsNLP34*	*MS.gene003861.t1*	chr4.2	38393971:38397944	915	101.99	5.16	Nucleus
*MsNLP35*	*MS.gene056603.t1*	chr4.3	36791789:36796033	914	101.81	5.13	Nucleus
*MsNLP36*	*MS.gene28321.t1*	chr4.4	39056054:39060302	915	101.9	5.16	Nucleus
*MsNLP37*	*MS.gene65320.t1*	chr5.2	14940801:14943763	312	36.46	8.2	Chloroplast, Nucleus
*MsNLP38*	*MS.gene65321.t1*	chr5.2	14973721:14976672	311	36.31	7.1	Chloroplast
*MsNLP39*	*MS.gene65377.t1*	chr5.2	83791572:83794736	932	102.54	5.99	Nucleus
*MsNLP40*	*MS.gene64875.t1*	chr5.3	13899347:13902296	312	36.47	7.69	Chloroplast, Nucleus
*MsNLP41*	*MS.gene64877.t1*	chr5.3	13944545:13947498	311	36.29	7.69	Chloroplast, Nucleus
*MsNLP42*	*MS.gene75795.t1*	chr5.3	80392837:80395995	932	102.57	5.99	Nucleus
*MsNLP43*	*MS.gene72645.t1*	chr5.4	15482882:15485832	312	36.39	6.74	Chloroplast, Nucleus
*MsNLP44*	*MS.gene72647.t1*	chr5.4	15507241:15510106	311	36.21	6.74	Chloroplast
*MsNLP45*	*MS.gene074493.t1*	chr5.4	78352417:78355564	927	102.01	5.97	Nucleus
*MsNLP46*	*MS.gene035459.t1*	chr6.1	33520669:33525989	857	96.19	5.41	Chloroplast, Nucleus
*MsNLP47*	*MS.gene30240.t1*	chr6.2	45235660:45241096	857	96	5.36	Chloroplast, Nucleus
*MsNLP48*	*MS.gene039361.t1*	chr6.3	43615999:43621488	857	96.15	5.34	Chloroplast, Nucleus
*MsNLP49*	*MS.gene09415.t1*	chr6.4	22166009:22171433	857	96.04	5.36	Chloroplast, Nucleus
*MsNLP50*	*MS.gene061721.t1*	chr8.1	16687283:16688427	246	29.09	5.37	Chloroplas, Cytoplasm, Nucleus
*MsNLP51*	*MS.gene033121.t1*	chr8.2	17306580:17307819	210	24.58	4.94	Chloroplast
*MsNLP52*	*MS.gene58429.t1*	chr8.3	15880487:15881726	210	24.58	4.94	Chloroplast
*MsNLP53*	*MS.gene044384.t1*	chr8.4	18676534:18677674	246	29.08	5.37	Chloroplast, Cytoplasm, Nucleus

### Multiple sequence alignment and phylogenetic analysis of the MsNLP genes

The phylogenetic relationship of *MsNLP*s was examined via multiple sequence alignment of their RWP-RK and PB1 domains in alfalfa and Arabidopsis proteins. Through a SMART protein domain query, it was found that the alfalfa NLP contains two typical RWP-RK and PB1 domains. The RWP-PK domain is highly conserved and consists of approximately 50 amino acids, and all *MsNLPs* contain “RWPXRK” characteristic residues (Additional file 2). The PB1 domain at the C-terminus is highly conserved and consists of approximately 80 amino acids. The 23 *MsNLPs* contained a complete PB1 domain, which was partially absent in other members.

**Fig. 1:**
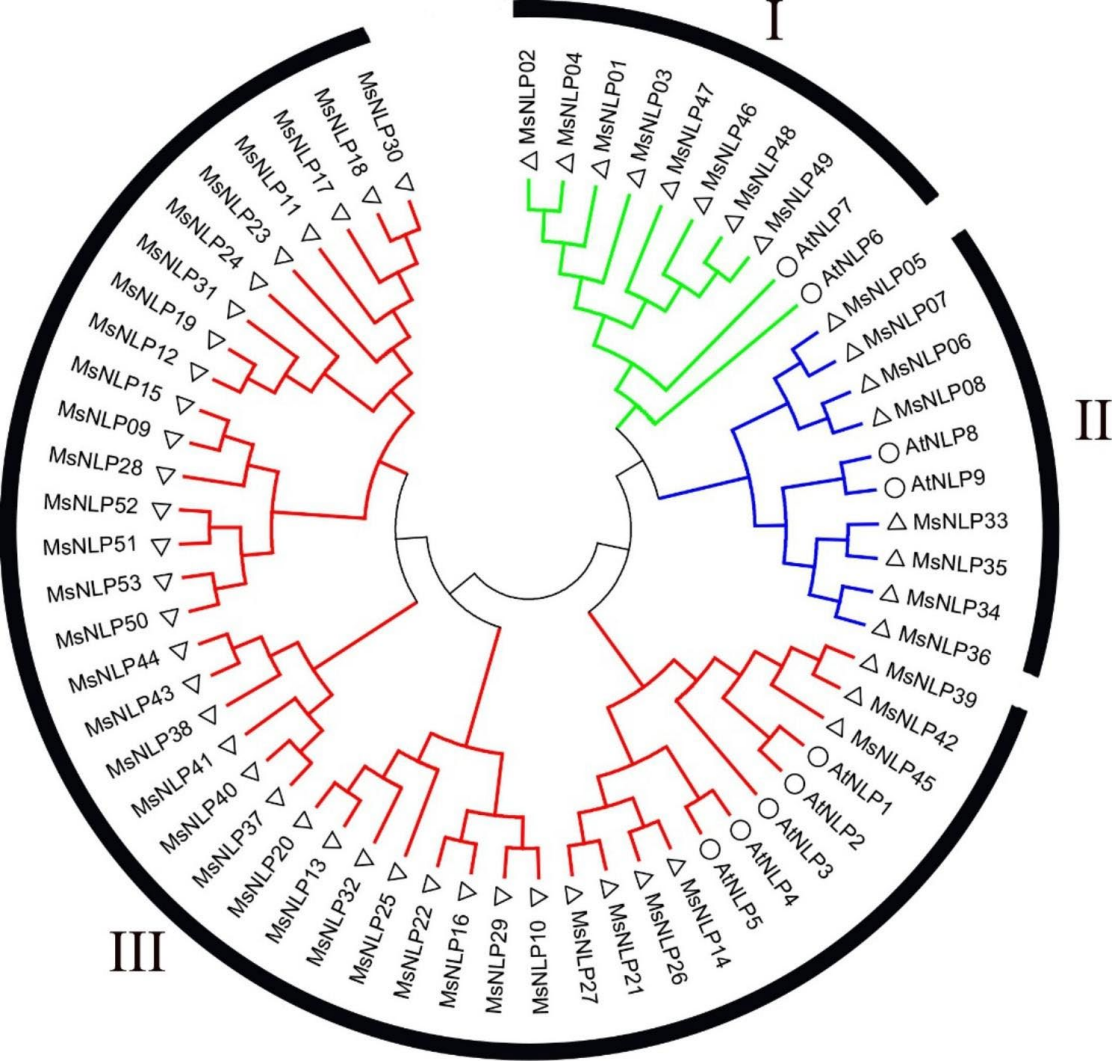
Phylogenetic analysis of NLP proteins in alfalfa and Arabidopsis. The three different colored arcs (red, green and blue) indicate different groups of NLP domains. The hollow triangles and circles represent NLP domains from alfalfa and Arabidopsis, respectively

To further explore the evolutionary relationships of the NLP gene family in alfalfa, the sequences of all 9 *AtNLPs* and 53 *MsNLPs* were used to construct a phylogenetic tree. Phylogenetic analysis indicated that the alfalfa NLP domains can be divided into three subgroups (named I to III), corresponding to the results of a previous study in Arabidopsis (Fig. 1). Both subclasses I and II contain 8 proteins, with each containing RWP-RK and PB1 domains, which is also the same as *AtNLP6/7* and *AtNLP8/9* proteins. Subclass III was the largest group, with the 37 remaining members having either both domains or only the RWP-RK domain.

### Gene structure and motif composition of the alfalfa NLP gene family

**Fig. 2:**
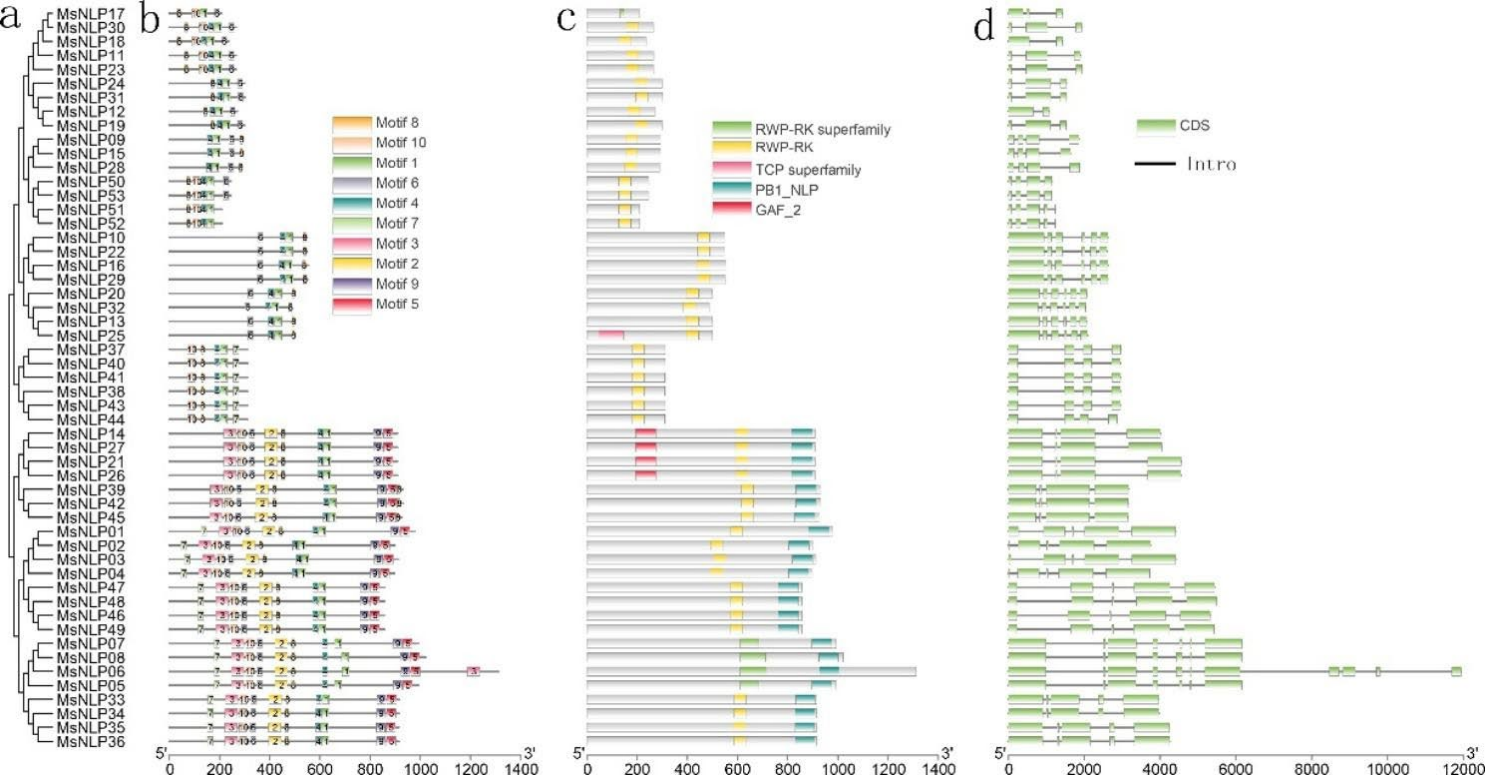
Phylogenetic relationships and structure of *NLP* genes from alfalfa and architecture of their conserved encoded protein motifs. **a** A phylogenetic tree was constructed by MEGA 6.0 using the neighbor-joining (NJ) method. **b** Motif composition of *NLPs* from alfalfa. The motifs, numbered 1–10, are displayed in different colored boxes. **c** Conserved motif analysis of alfalfa *NLP* genes. **d** Exon–intron structure of alfalfa *NLP* genes. The light green boxes indicate exons, and the black lines indicate introns

Motif divergence was examined to gain more insight into the evolution of the 53 *MsNLPs* [24]. A total of 10 motifs were predicted and used to analyze the features of the NLP gene family. The sequence information for each motif is provided in Additional file 3 and Additional file 4. As shown in Fig. 2b, each *MsNLP* contained between four and ten motifs; some motifs were shared by all members, while others existed in only a few subgroups. For example, motif 1 and motif 8 were present in all members, motif 4 was present in all members except *MsNLP17*, and motif 7 was detected in only 22 members. Motifs 1 and 8 represent the RWP-RK superfamily domain and RWP-RK domain, respectively. Motif 4 represented the PB1 domain, which was located at the C-terminus **(**Fig. 2c**)**. These results indicated that the RWP-RK domain and PB1 domain were conserved and specific to plant *NLPs*. The similar gene structures observed in the same *MsNLP* subfamily members are consistent with their phylogenetic relationships **(**Fig. 2a**)**.

The intron diversity divergence of proteins could also provide more insight into the evolution of the NLP gene family in alfalfa. As shown in Fig. 2d, the number of *MsNLPs* introns ranged from 1 to 10 (2 with one intron, 7 with two introns, 18 with three introns, 14 with four introns, 8 with five introns, 3 with six introns, and 1 with ten introns). The closely related members of each cluster have similar intron structures and small differences in length. Overall, the similarity of conserved motif composition and gene structure of the NLP members in the same groups, combined with the results of phylogenetic analysis, could strongly confirm that the phylogenetic classification was reliable.

### Synteny analysis and evolutionary selection pressure of the MsNLP genes

**Fig. 3:**
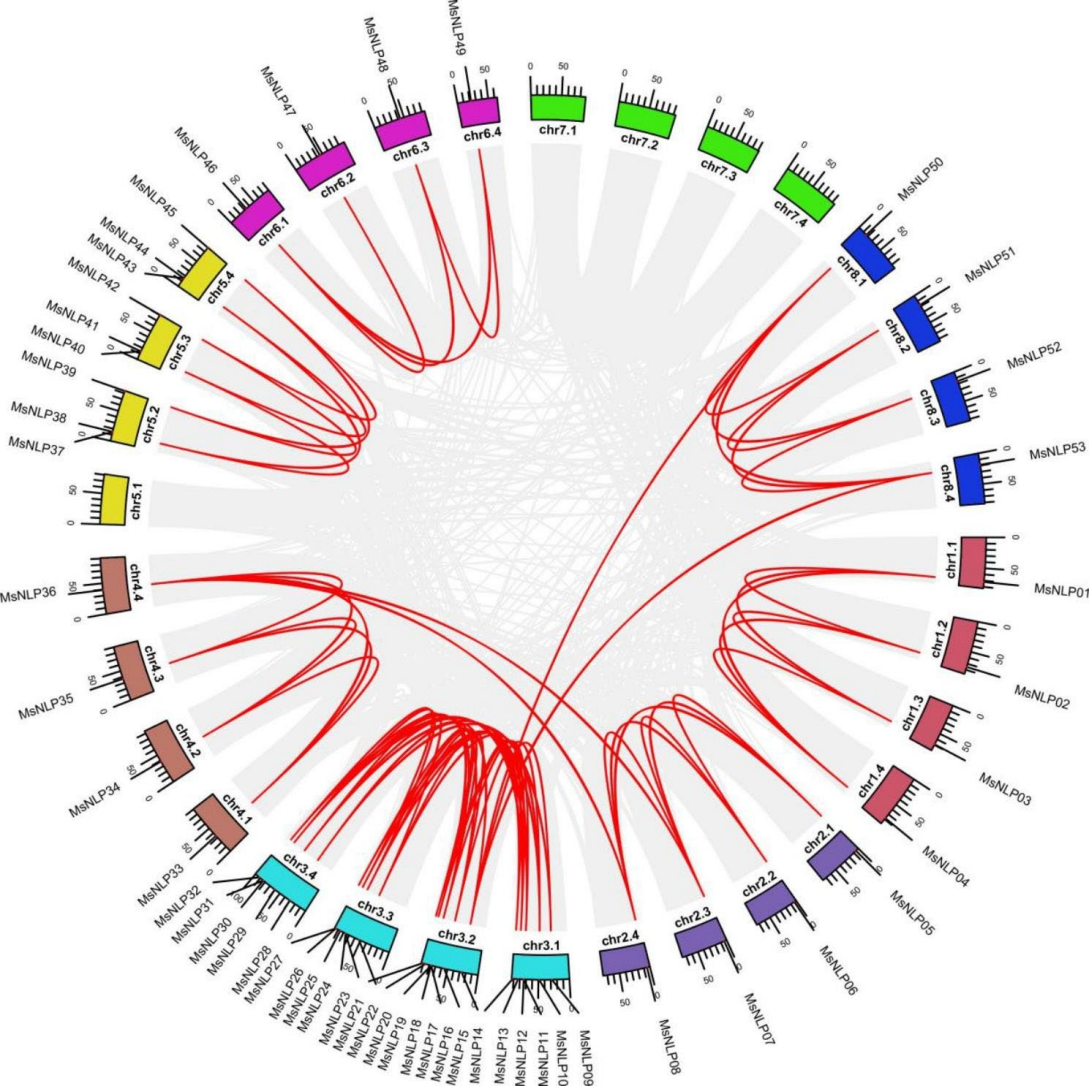
Synteny analysis of *NLP* genes in alfalfa. The gray lines indicate all synteny blocks in the alfalfa genome, and the red lines indicate duplicated *NLP* gene pairs

To increase the knowledge of the functions of and mechanisms underlying of NLP gene family members in alfalfa, fragment replication event were investigated. As shown in Fig. 3, four gene pairs (*MsNLP7*/*36*, *MsNLP8*/*36*, *MsNLP15*/*50* and *MsNLP15*/*53*) were distributed on different chromosomes in 69 collinear gene pairs and were considered fragment duplication genes (Additional file 5). These results indicated that *MsNLP* genes probably originated via gene duplication and that segmental duplication events played a major driving force in *MsNLP* evolution.

**Fig. 4:**
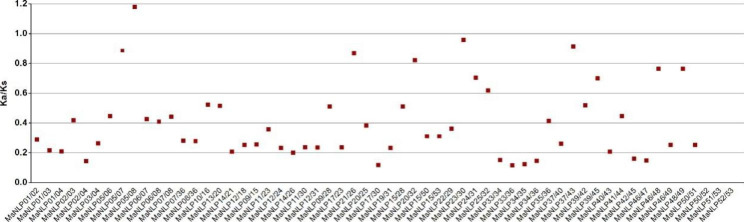
The Ka/Ks ratios of *MsNLP* collinear gene pairs in alfalfa. The abscissa represents different gene pairs, and the ordinate represents the ratio of Ka/Ks.

To further analyze the selective constraints among the *NLP* genes in alfalfa, we calculated the Ka/Ks ratio for the *NLP* gene pairs. Ka mutations are subject to natural selection, while Ks mutations are not. Neutral selection has occurred when the Ka/Ks ratio equals 1, a Ka/Ks ratio greater than 1 indicates positive selection, while a Ka/Ks ratio less than 1 indicates purifying selection. The distributions of Ka and Ks between each *MsNLP* pair are shown in Fig. 4. The Ka of *MsNLP* ranged from 0.001374 to 0.378740, while the Ks ranged from 0.001635 to 1.21454. Among all collinear gene pairs, most pairs for which the Ka/Ks ratio was < 1 were subjected to negative selection, whereas only one was subjected to positive selection (Additional file 6). Taken together, these results suggested that *MsNLP* genes primarily underwent negative selection; that is, they were subject to purifying selection during evolution.

### Cis-acting elements in alfalfa NLP genes

**Fig. 5:**
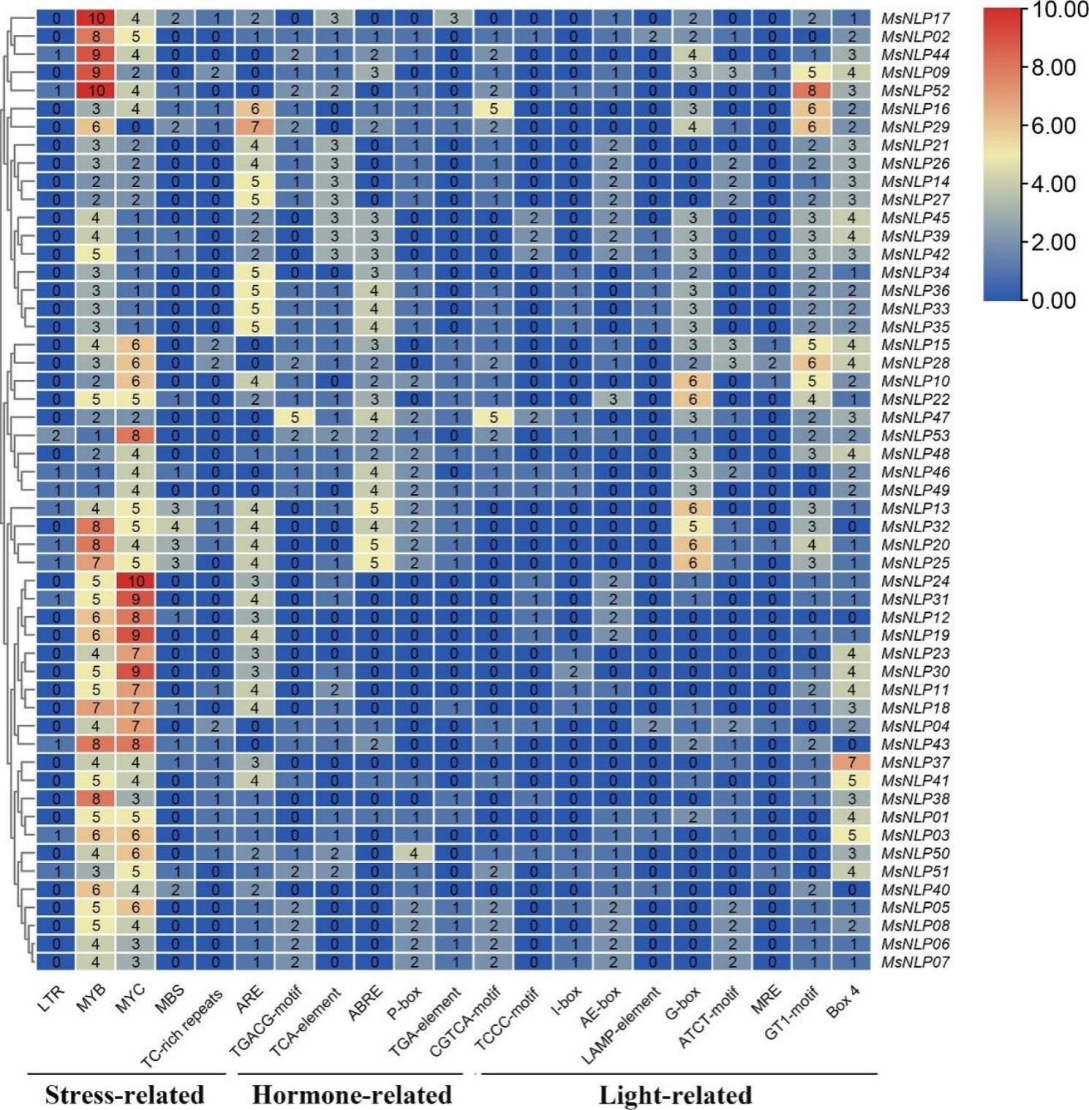
Predicted cis-acting elements within the promoters of *NLP* family members in alfalfa. The 2.0 kb regulatory region upstream of ATG was analyzed with PlantCARE software. The red represents high expression levels, and the blue represents low expression levels

To further explore the potential function of *MsNLP* genes, the 2000 bp regulatory region upstream of the promoter was selected to analyze the cis-acting elements. Stress-, hormone- and light-responsive elements were detected in the promoter regions of NLP family genes in alfalfa (Fig. 5). Many cis-acting elements related to stress responses, such as LTRs (13), MYBs (254), MYCs (246), MBSs (30), TC-rich repeats (22), and AREs (130), were found in the promoter regions of *NLP* genes. Hormone-responsive elements such as TGACG motifs (43), TCA elements (54), ABREs (83), P-boxes (49), TGA elements (23), and CGTCA motifs (49) were detected in the promoter regions of *MsNLP* genes. Moreover, light-responsive elements, for instance, TCCC motifs (18), I-boxes (19), AE-boxes (46), LAMP elements (13), G-boxes (100), ATCT motifs (38), MREs (8), GT1 motifs (110), and Box 4 elements (120), were also found in the promoter of *MsNLP* genes. Taken together, these results indicated that *MsNLP* genes may be regulated by cis-acting elements within their promoters during alfalfa growth and in response to stress and phytohormone.

### Tissue-specific expression of MsNLPs

**Fig. 6:**
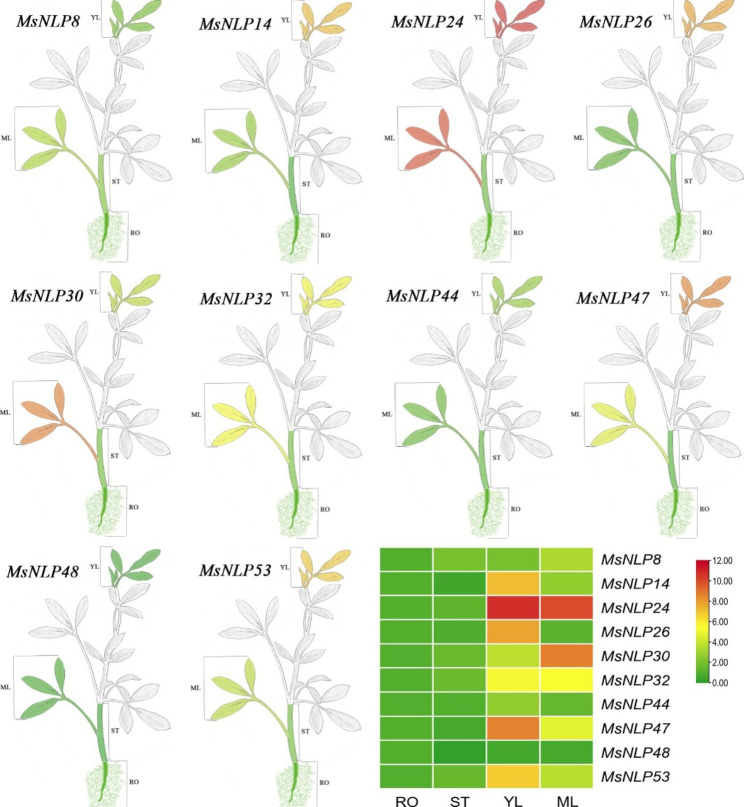
Differential expression of representative *MsNLP* genes in different tissues, as determined by qRT-PCR. RO: roots; ST: stems; YL: young leaves; MF: mature leaves. The mean expression value was calculated from three independent biological replicates and is expressed in relate to that in the roots. The red represents high expression levels, and the green represents low expression levels

To gain a more in-depth understanding of the function of *NLP* genes in alfalfa development, 10 *MsNLPs* were randomly selected to analyze their expression in four different tissues (roots, stems, young leaves and mature leaves) via qRT-PCR (Fig. 6). Almost all the genes exhibited tissue-specific transcript accumulation patterns, while the expression profiles of only one gene (*MsNLP48*) were similar in different tissues. The expression levels of all selected *MsNLPs* (except *MsNLP48*) were higher in the leaves (including both young leaves and mature leaves) than in the roots and stems. *MsNLP14*/*24*/*26*/*44*/*47*/*53* were highly expressed in young leaves, and *MsNLP*8/30/32 were highly expressed in mature leaves. The *MsNLP* gene was obviously expressed specifically in the leaves, which indicated that it might be involved in leaf growth and development.

### Expression of MsNLPs in response to abiotic stress in leaves

**Fig. 7:**
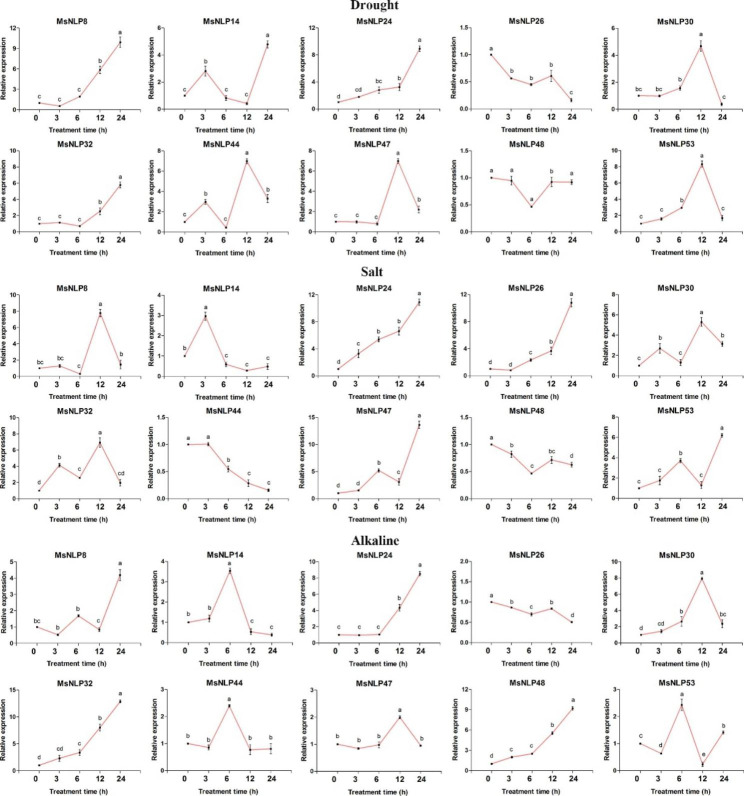
Expression profiles of 10 selected *MsNLP* genes in response to different stress treatments. The data were normalized to those of the *GAPDH* gene. The mean expression values were calculated from three independent biological replicates and are relative to those of the 0 h controls. The different letters indicate that the mean values are significantly different among the treatments (α = 0.05)

To explore the potential functions of the *MsNLP* genes in response to different abiotic stresses, the expression profiles of 10 *MsNLP* genes in response to drought, salt and alkaline stresses were investigated by qRT‒PCR analysis (Fig. 7). The expression levels of some genes tended to constantly fluctuate, which increased in the early stage and then continuously decreased, as was the case for the *MsNLP14* gene in response to salt stress. Most genes were expressed at extremely high levels at 12 and 24 h. In general, all *MsNLPs* were expressed in response to all abiotic treatments. Some genes could be induced by multiple stresses; for example, *MsNLP24* was upregulated within 24 h of all tested treatments. Some genes were upregulated until 12 h and then downregulated continuously, as was the case for *MsNLP30*/*47* in response to alkaline stress, whereas other genes were not expressed for 3 h but were upregulated thereafter, as was the case for *MsNLP8* in response to drought stress, suggesting that these *MsNLP* genes might play a crucial role at early and later stages. In addition, some *MsNLP* genes exhibited opposite expression patterns under different stress conditions. For instance, *MsNLP26* was upregulated continuously within 24 h of salt stress treatment but exhibited the opposite expression pattern in response to drought and alkaline stress. In conclusion, *MsNLP* genes were significantly induced or repressed by the above three treatments.

### MsNLP genes expressed in response to exogenous phytohormones

**Fig. 8:**
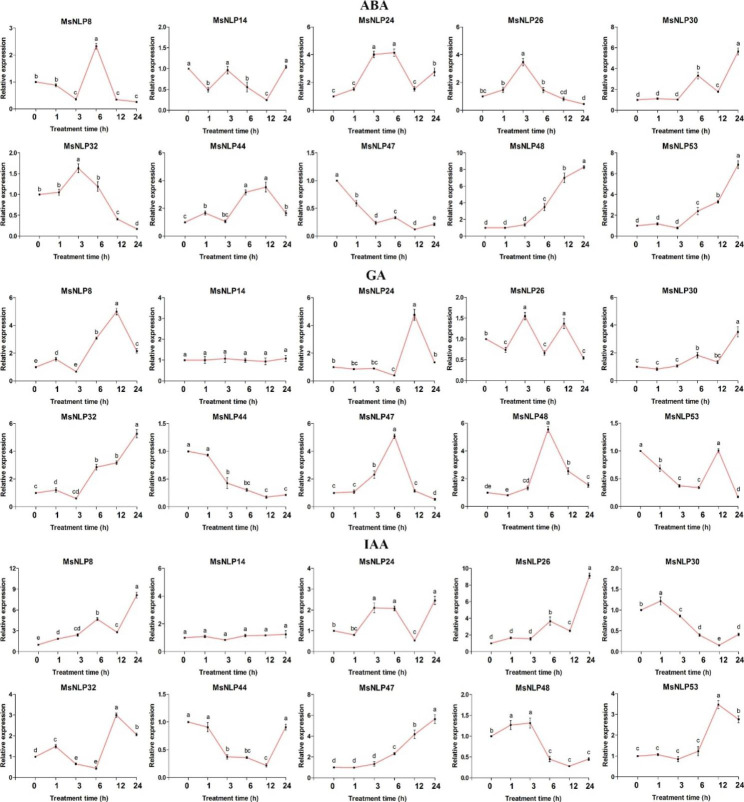
Expression profiles of 10 selected *MsNLP* genes in response to different phytohormone treatments. The data were normalized to those of the *GAPDH* gene. The mean expression values were calculated from three independent biological replicates and are relative to those of the 0 h controls. The different letters indicate that the mean values are significantly different among the treatments (α = 0.05)

To confirm whether MsNLP family genes were influenced by different phytohormone treatments, qRT‒PCR experiments were performed to measure the expression levels of *MsNLPs* in response to treatment with different phytohormones, including abscisic acid (ABA), gibberellin (GA) and indoleacetic acid (IAA) (Fig. 8). Overall, most *MsNLP* genes were induced by at least one phytohormone treatment; for example, *MsNLP 8*/*24*/*26*/*30*/*44*/*47*/*48*/*53* were induced in response to all tested treatments. In contrast, some *MsNLP* genes were induced in response to only one or two treatments. For instance, *MsNLP14* expression was induced in response to only ABA treatment. Among these *MsNLP* genes, the expression of some was not significantly changed within 3 h and increased in the later period after phytohormone treatment, as was the case for *MsNLP48* and *MsNLP53* in response to ABA, so they may be related to later responses to phytohormone treatments; on the other hand, *MsNLP48* was upregulated at 3 h and then decreased after IAA treatment, after which its expression was not significantly changed until 24 h, suggesting that this *MsNLP* gene plays an important role at the early stage of the response to exogenous phytohormones. Interestingly, the expression patterns of the *MsNLP* genes were opposite in response to phytohormone treatments. For instance, *MsNLP47* was significantly induced within 24 h of IAA treatment but was repressed by ABA treatment.

## Discussion

In this study, a total of 53 *NLP* genes were identified and sequentially named *MsNLP1* to *MsNLP53* on the basis of their chromosomal location in alfalfa. Previous studies have revealed nine NLP family genes in Arabidopsis, six in rice and nine in maize [3, 5, 11], indicating that different species have different numbers of NLPs. NLP family genes encode large proteins with slightly relatively low pI values in alfalfa (Table 1), consistent with results of other species, which indicates that these TFs are preferentially active under acidic conditions [5, 7, 25]. All *MsNLPs* identified in this study contain RWP-RK domains; however, only 23 members contain an intact PB1 domain, and the PB1 domain of the rest is partially deleted (Additional file 2), which was caused by sequence fragmentation during evolution or a sequencing error in the alfalfa genome [12]. Therefore, domain gain and loss are divergent forces that drive expansion of the NLP gene family [26, 27].

Phylogenetic analysis indicated that the *NLP* genes can be divided into three groups in alfalfa (Fig. 1), which is consistent with previous evolutionary classifications in Arabidopsis, rice and maize. The number of *MsNLP* introns ranged from 1 to 10 in alfalfa, which was consistent with the results found in Arabidopsis, indicating that different species display similarities in terms of the diversity of the structure of the *NLP* genes [5]. Previous research has shown that segmental duplication can result in a slower rate of intron gain than intron loss [28]. Group II had more introns than group I and group III (Fig. 2), indicating that group II was the most conserved because this group might contain the original genes. Overall, the similarity of conserved motif composition and gene structure of the NLP members in the same groups, combined with the results of phylogenetic analysis, strongly confirm that the phylogenetic classification was reliable. Segmental duplication events are critical for the rapid expansion and evolution of gene families and are essential for environmental adaptability and speciation [29–31]. According to our collinearity analysis of the *MsNLP* genes, we observed all pairs of four groups of fragment duplications (Fig. 3and Additional file 5), in which similar events have been found to have occurred in many species, including Arabidopsis thaliana, rice [5], tomato [32] and others. Therefore, the presence of duplication events implied that segmental duplications were the major evolutionary mechanisms that drove alfalfa *NLP* expansion in our research. Notably, the Ka/Ks ratio for all the *NLP* gene pairs except one (*MsNLP05*/*08*, which was greater than 1) was less than 1 (Fig. 4and Additional file 6), implicating mainly purifying selection, which favours the elimination of deleterious mutant genes during evolution [33]. This result is consistent with the study of the *NLP* gene family in maize [3].

Tissue-specific expression patterns of *NLP* genes in different tissues have been investigated in several plant species. For example, by assessing their expression levels in different tissues of Arabidopsis and rice, researchers have found that *NLPs* are expressed widely in almost all organs, including seeds, roots, stems, flowers, leaves, nodules, inflorescences, etc [7]. In Arabidopsis, *AtNLP8* and *AtNLP9* are highly expressed in senescing leaves and seeds compared with other organs, and their expression levels are moderate or very low. *OsNLP1* and *OsNLP3*, whose transcripts constitute the majority of OsNLP transcripts, were found to be preferentially expressed in source organs [5]. It has also been reported that *NLPs* are highly expressed in the leaves, roots, male catkins, xylem, seeds, and female catkins of *Brassica napus* [12]. In the present study, the expression levels of most genes except *MsNLP48* were higher in the leaves than in the roots and stems. *MsNLP14*/*24*/*26*/*44*/*47*/*53* were highly expressed in young leaves, whereas *MsNLP8*/*30*/*32* were highly expressed in mature leaves (Fig. 6), indicating that these genes may participate in leaf growth and development. In addition, we speculate that *MsNLPs* accumulate in leaves might be used for storing nitrogen to coordinate leaf expansion and photosynthetic capacity to promote leaf growth and biomass, according to previous reports [12, 34]. Moreover, the expression of alfalfa *NLPs* in the roots was much lower than that in the leaf tissues (in which expression levels were high), indicating that *NLPs* are involved in mainly nitrate transport rather than the nitrate absorption process [11].

NLPs are essential TFs involved in nitrate signalling [35–37]. It has been reported that nitrate triggers nitrate-CPK (Ca^2+^-sensor protein kinase)-NLP signalling and nitrate-coupled CPK signalling to phosphorylate NLPs, which play a central role in nitrate signalling and integrate transcription, transport, metabolism and systematic growth processes in plants [14, 17, 38]. In our study, analysis of the promoters of the *NLP* gene family members revealed many cis-acting elements associated with stress- and hormone-responsive elements, suggesting that the *MsNLP* genes may be regulated by cis-acting elements within their promoters during alfalfa growth and in response to stress and phytohormones (Fig. 5). It has been shown that *OsNLP4* is repressed by several abiotic stresses, including drought, cold and submergence in *ric*e [39]. *OsNLP3* is induced after germination and repressed by heat treatment [40]. Previous studies have shown that *Arabidopsis thaliana* lacking *AtNLP7* is more drought resistant than the normal wild type, suggesting that *NLP* expression affects plant tolerance to drought [17]. The expression of *SlNLP1*, *SlNLP2*, *SlNLP4*, and *SlNLP6* was upregulated after nitrogen starvation treatment in tomato [41]. *OsNLP1*, a key gene regulating nitrogen utilization, was rapidly induced by nitrogen starvation in rice [42]. In addition, it was shown that *nlp7* mutant plants were hypersensitive to ABA and submergence-induced hypoxia in *Arabidopsis thaliana* [43]. The expression of *LATD/NIP* is regulated by hormones, particularly by abscisic acid, which has been previously shown to rescue the primary and lateral root meristem arrest of latd mutants in *Medicago truncatula* [44]. These results are consistent with the present study, in which *MsNLPs* were equally responsive to abiotic stresses and hormones in alfalfa. Therefore, the goal of this study was to obtain further insights into the biological functions of the *MsNLP* genes in response to abiotic stress and phytohormone treatment. Previous research has shown that abiotic stress not only affects alfalfa growth and development but also usually leads to decreases in crop yield and quality [45]. Our results revealed that ten *NLP* genes were upregulated or downregulated under drought stress, especially at 12 h of prolonged stress (Fig. 7). Combined with the results of previous research that Arabidopsis thaliana *AtNLP7* knockout plants were more drought resistant than wild-type plants, the results of the present study indicate that NLP TFs are related to drought resistance in plants [17]. Although *MsNLPs* exhibited different expression patterns, *MsNLPs* were expressed in response to both salt and alkaline treatment. In addition, the expression of most *MsNLP* genes significantly decreased or increased following ABA treatment (Fig. 8). In Arabidopsis, *AtNLP8* can directly bind to the promoter of an ABA catabolism-related enzyme-encoding gene to reduce ABA levels, which implies that NLP TFs may participate in the regulation of the ABA signalling pathway. Similarly, some *MsNLP* genes were induced by GA and IAA phytohormone treatment. Overall, our study lays a foundation for further functional investigation of NLPs in alfalfa.

## Conclusions

In summary, 53 *NLP* genes were identified and further classified into three main groups in alfalfa, with highly conserved intron structures and motif compositions occurring within members of the same group. Synteny analysis and the Ka/Ks ratios revealed that the *MsNLP* genes underwent fragment duplication events and were subjected to purifying selection during evolution. Tissue expression pattern and expression profile analyses indicated that the *MsNLP* genes play pivotal roles in alfalfa development and participate in the response to abiotic stress and phytohormone-related signal transduction processes. These results provide a valuable resource for an improved understanding of the characteristics and biological roles of the *MsNLP* genes in alfalfa.

## Materials and methods

### Identification and characterization of alfalfa NLP genes

First, the protein sequences of the 9 NLP members of Arabidopsis thaliana were downloaded from a database (http://www.arabidopsis.org) and used as query sequences. Afterward, the published whole-genome protein sequence of cultivated alfalfa (cultivated XinJiangDaYe ) were used to construct a library of the genome database (https://figshare.com/projects/whole_genome_sequencing_and_assembly_of_Medicago_sativa/66380), and the alfalfa genome sequence was then subjected to local BLASTP searches, where the E value was than 1e-5. Subsequently, in accordance with the hidden Markov model (HMM) query strategy, the sequence of the conserved RWP-RK (PF02042) domain of the NLP gene family members was downloaded from the Pfam (http://pfam.xfam.org/) database, after which HMMER 3.0 software was used to search the alfalfa genome and filter genes that were 100% similar and whose E value was less than 1e-5. Finally, after the removal of duplicates and integration of the alfalfa NLP sequence information obtained via the above two procedures, each NLP member was named sequentially according to its distribution on the chromosomes of alfalfa [46]. Physicochemical properties such as the length, molecular weight (MW) and isoelectric point (pI) of the proteins were predicted using the ExPASy ProtParam (www.expasy.org) online analysis tool. In addition, the subcellular locations of alfalfa NLP proteins were predicted by WoLF PSORT (http://www.csbio.sjtu.edu.cn/bioinf/Cell-PLoc-2/).

### Multiple sequence alignment and phylogenetic analysis

The domain sequences of the characterized NLP proteins were used to create multiple protein sequence alignments using ClustalW, with the default parameters [47]. Phylogenetic analysis was performed with MEGA 6.0 using the maximum likelihood method with the following parameters: Poisson model, pairwise deletion, and 1000 bootstrap replications [48].

### Gene structure and motif composition of alfalfa NLP gene family members

Conserved domains were analyzed and visualized using CD-Search (https://www.ncbi.nlm.nih.gov/Structure/cdd) [49, 50]. The conserved motif sequences of the genes were subsequently analyzed by the MEME program (http://meme-suite.org/), and the structure of the *MsNLP* genes was analyzed with TBtools software [51].

### Synteny analysis and evolutionary selection pressure of MsNLP genes

To analyze the syntenic relationship of *MsNLP* genes, syntenic analysis maps were constructed using the MCScanX software tool. Based on the synteny map of the *MsNLP* genes, nonsynonymous (Ka) and synonymous (Ks) nucleotide substitutions of each duplicated *NLP* gene were calculated using KaKs_Calculator 2.0 [52].

### Analysis of cis-element within the MsNLP gene promoter

The *NLP* gene translation start site approximately 2000 bp upstream of ATG from the alfalfa genome was selected as the promoter region, and all the *MsNLP* promoter sequences were submitted to PlantCARE (http://bioinformatics.psb.ugent.be) to search for plausible cis-acting elements [53].

### Plant materials and treatments

Cultivated *Medicago sativa* L. ‘XinjiangDaYe’, provided by the Xinjiang Academy of Agricultural Sciences, was used in this study. Alfalfa seeds with full grains and consistent morphology were selected for cultivation in vermiculite, and 1/10-strength Hoagland nutrient solution was applied through irrigation during this period. Vigorously growing alfalfa plants were selected for determination of tissue expression specificity and expression patterns in response to various stresses and hormone treatment experiments at 30 d after sowing. The stems, roots, young leaves and mature leaves of alfalfa plants were collected separately for RNA extraction and subjected to quantitative real-time PCR (qRT-PCR) analysis. For drought, salt and alkaline treatments, the plants were subjected to 15% polyethylene glycol (PEG) 6000, 150 mM NaCl and 150 mM NaHCO_3_ for 3, 6, 12 and 24 h. For phytohormone treatments, 30-d-old seedlings were transplanted into Murashige and Skoog (MS) medium liquid media supplemented with 100 µM ABA, 100 µM GA and 100 µM IAA for 1, 3, 6, 12 and 24 h. Three independent replicates were included for each treatment, and the tissues were collected, immediately frozen in liquid nitrogen and stored at − 80 °C for subsequent analysis.

### RNA extraction and gene expression analysis

An Ultrapure RNA Kit (CoWin Biotech, Beijing, China) was used to extract the total RNA from the samples according to the protocol provided by the manufacturer. In accordance with the HiScript II Q Select Reverse Transcriptase Kit (Vazyme Biotech, Nanjing, China) instructions, oligo (dT) 10 reverse primers were used for first-strand cDNA synthesis. Quantitative RT-PCR was carried out according to the method provided by ChamQ™ Universal SYBR qPCR Master Mix (Vazyme, China). The alfalfa *GAPDH* gene was used as an internal control, and all the primers used are listed in Additional file 7. The reaction was carried out as follows: 95 °C for 30 s; 40 cycles of 95 °C for 5 s and 60 °C for 34 s; and 95 °C for 15 s [54, 55].

### Statistical analyses

Each experiment included three independent biological replicates and technical replicates, and all the data are expressed as the means ± standard deviations (SDs). GraphPad Prism 5.0 was used to map the final data, and differences among groups were tested using one-way ANOVA with SPSS 25.0 software. Relative gene expression levels were evaluated according to the 2 ^−ΔΔCT^ method.

## Electronic supplementary material

Below is the link to the electronic supplementary material.

Supplementary Material 1

Supplementary Material 2

Supplementary Material 3

Supplementary Material 4

Supplementary Material 5

Supplementary Material 6

Supplementary Material 7

## Data Availability

The data does not involve sequencing and are listed in the article and its additional files.

## Abbreviations

ABA: Abscisic acid
GA: Gibberellin
GAF: cGMP phosphodiesterase
HMM: Hidden Markov model
IAA: Indoleacetic acid
Ka: Nonsynonymous substitution ratio
Ks: Synonymous substitution ratio
MS: Murashige and Skoog
MW: Molecular weight
NJ: Neighbor-joining
NLP: NIN-LIKE PROTEIN
PEG: Polyethylene glycol
pI: Isoelectric point
qRT-PCR: Quantitative real-time PCR
TFs: Transcription factors
