# Supplementary figures and images for: Genome-wide investigation of NLP gene family members in alfalfa (Medicago sativa L.): evolution and expression profiles during development and stress
Source: BMC Genomics. 2023 Jun 13;24:320. doi: 10.1186/s12864-023-09418-x (PMC10262365; doi:10.1186/s12864-023-09418-x)

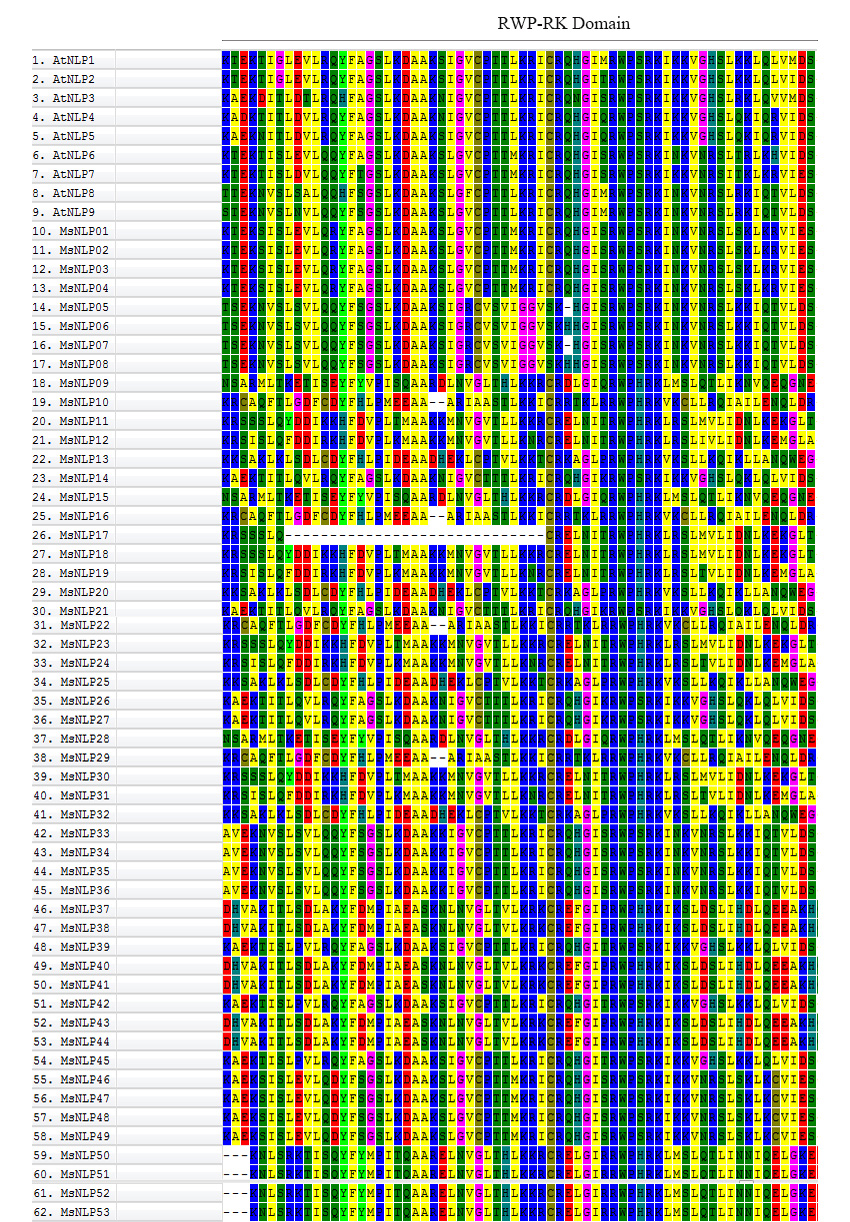

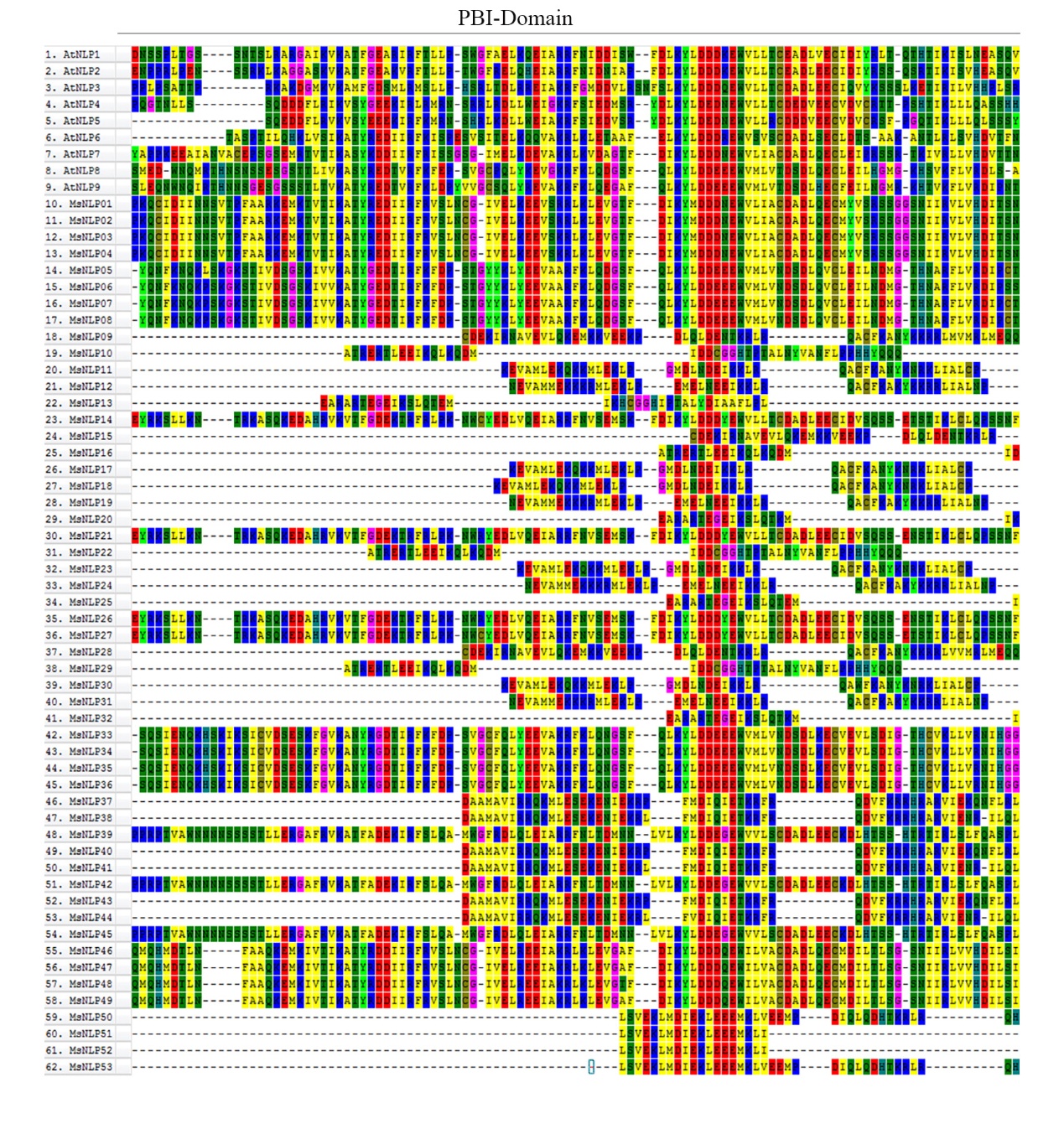
**Additional file 2** RWP-RK and PB1 domains

Supplement: Supplementary file 2 — Supplementary Material 2 [file 12864_2023_9418_MOESM2_ESM.docx]
