# Supplementary material for: Genome-wide investigation of NLP gene family members in alfalfa (Medicago sativa L.): evolution and expression profiles during development and stress
Source: BMC Genomics. 2023 Jun 13;24:320. doi: 10.1186/s12864-023-09418-x (PMC10262365; doi:10.1186/s12864-023-09418-x)
Supplement: Supplementary file 7 — Supplementary Material 7 [file 12864_2023_9418_MOESM7_ESM.docx]

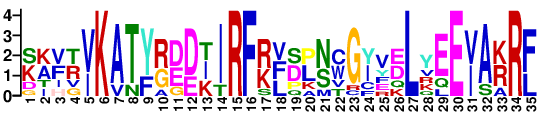


**Motif 1**


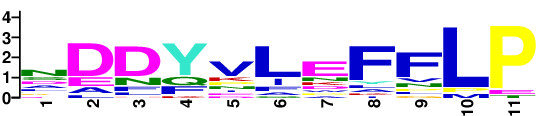


**Motif 2**


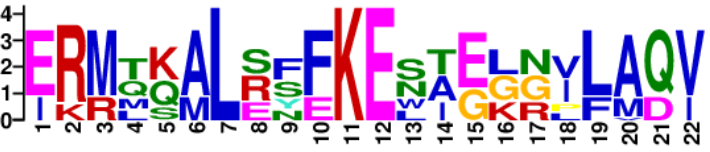


**Motif 3**


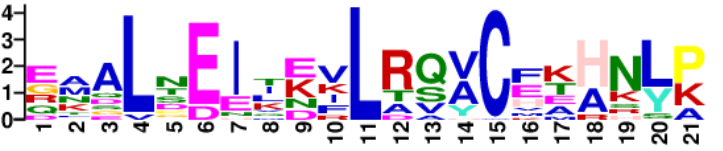


**Motif 4**


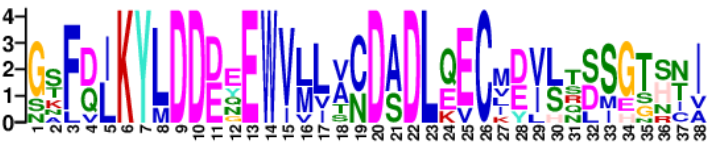


**Motif 5**


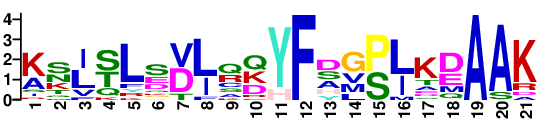


**Motif 6**


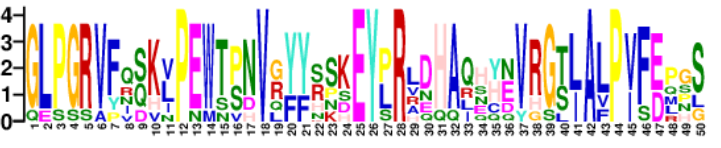


**Motif 7**


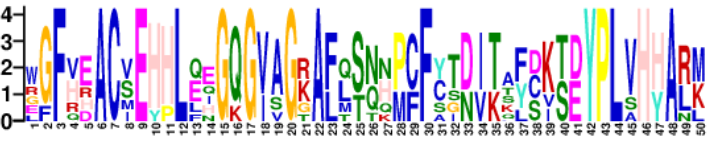


**Motif 8**


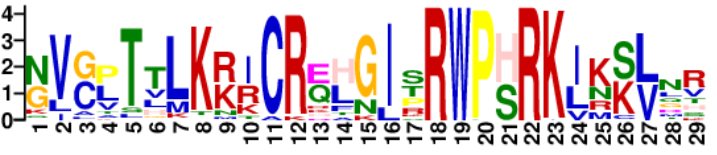


**Motif 9**


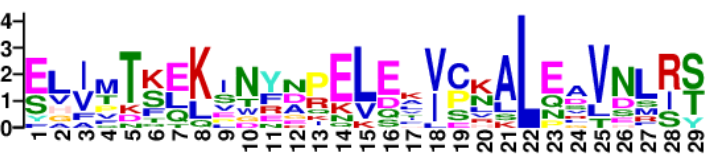
**Motif 10**

**Additional file 3** Information of10motifs.
